# Smart License Plate in Combination with Fluorescent Concentrator for Vehicular Visible Light Communication System

**DOI:** 10.3390/s22072485

**Published:** 2022-03-24

**Authors:** Seoyeon Oh, Yejin Lee, Minseok Yu, Seonghyeon Cho, Sana Javed, Hyunchae Chun

**Affiliations:** Department of Information and Telecommunication Engineering, Incheon National University, Incheon 22012, Korea; seoyeon.oh@inu.ac.kr (S.O.); vexia@inu.ac.kr (Y.L.); alseoddl852@inu.ac.kr (M.Y.); seonghyeon.cho@inu.ac.kr (S.C.); sanajaved256@gmail.com (S.J.)

**Keywords:** Li-Fi, visible light communication, VLC, optical wireless communication, OWC, vehicle-to-vehicle communication, V2V

## Abstract

Vehicle-to-vehicle communication based on visible light communication has gained much attention. This work proposes a smart license plate receiver incorporated with a fluorescent concentrator, enabling a fast vehicle-to-vehicle communication with a large field of view and high optical gain. Communication performance is experimentally analyzed using off-the-shelf light-emitting diode-based headlamps for low-latency direct line of sight channel. Additionally, a blue laser diode-based beam-steering and tracking system, through image processing of taillights with a steerable mirror, is investigated. Data rates of 54 Mbps from the headlamps and 532 Mbps from the beam-steering channel with ±25° are demonstrated. In addition, real-time video streaming through the beam-steering channel is presented.

## 1. Introduction

Recently, the progress of autonomous driving has received great attention from both academia and industry. As research on this technology continues, the development of cooperative intelligent transportation systems (C-ITS) is considered crucial [1]. Vehicle-to-everything (V2X), a core of C-ITS, is a communication technology enabling information sharing among infrastructures and vehicles on the road through wireless networks. V2X mainly consists of vehicle-to-vehicle (V2V), vehicle-to-infrastructure (V2I), and vehicle-to-pedestrian (V2P) communications. In particular, V2V allows fast and direct exchange of information about neighboring vehicles, such as the position, speed, sensor information, and real-time black-box video. Therefore, it provides the functions of safety, security, and the avoidance of traffic congestion [2].

Many V2V studies have been conducted worldwide for better autonomous driving. In reference [3], a new cooperative transmission scheme called cooperative superposed transmission (CST) was suggested to ensure high reliability in various road scenarios. Additionally, a control system maintaining faster response and better stability has been introduced in the communication link between V2V- and non-V2V-equipped vehicles [4]. With the recent popularity of machine learning, the use of a long short-term memory (LSTM) neural network was also proposed to solve the transmission latency of the network between vehicles [5].

Visible light communication (VLC) is considered a candidate to supplement the intelligent transportation system (ITS) with the recent development of high-intensity light-emitting diode (LED) devices. Since LED lamps are already widely used in vehicles and roadside units, an active investigation on V2V-VLC utilizing such optical resources is ongoing globally [6,7,8,9]. The impact of line-of-sight (LOS) and non-LOS (NLOS) channels on communication performance was simulated in Ref. [6]. Since weather condition is one of the key factors that greatly influence communication using visible light, Fresnel lens and red LED with multiple Tx/Rx system was implemented to minimize the detrimental impact in Refs. [7,8]. To mitigate the effect of sunlight, a method of moving transmitter (Tx) and receiver (Rx) adaptively to the ambient light was introduced [9]. However, there are still unresolved challenges left for the development of V2V-VLC technology. Among them are the narrow field of view (FoV), LOS blockage, and misalignment. Typical VLC receivers require optics such as lenses or concentrators, leading to the limited FoV. Even within the FoV, the V2V channel between two vehicles is susceptible to another vehicle intervening in-between and by a vibrational and angular motion of the end device.

A fluorescent concentrator (FC) is a promising solution for enhancing the FoV of V2V-VLC system. FC is a non-imaging optic that accepts the incident photons from wide angles, converts them into longer wavelength photons, and guides them to a narrow edge through the total internal reflection [10]. Hence, a large FoV and high optical gain beyond the Étendue limit is provided, with the gain of ~4 compared to the conventional concentrators [11]. Additionally, with a short fluorescent lifetime, FC can accommodate a high bandwidth signal, overall improving the signal-to-noise ratio (SNR) in a wide frequency range.

For the link disconnection by the LOS blockage and the misalignment, a promising solution is to construct a beam-steering network with the surrounding vehicles equipped with a stable tracking method. To show the feasibility of the beam-steering technique in V2V-VLC, an onboard sensors architecture and cooperative data sharing between vehicles to estimate the pointing directions were studied in Ref. [12]. Additionally, a cost-effective beam-steering system based on a fluorescent reflector and image processing was introduced for device-to-device (D2D) V2V [13].

In this research, FC-coupled license plate with a beam-steering-technique-based V2V-VLC system is proposed. The unique features of FC (high gain and wide FoV) are tested under various practical cases. Off-the-shelf white LED (WLED) headlamps to construct a direct communication link and a narrow-beam blue laser diode (LD) channel to the FC-coupled license plate is evaluated. The position information obtained by two cameras beside the LD beam-steering module is utilized for the beam steering. With DC biased OFDM (DCO-OFDM) scheme, the communication performances of the onboard WLED channel and the beam-steered blue LD channel are investigated.

The rest of this work is organized as follows. Section 2 presents a description of the proposed V2V-VLC architecture, including headlamps, taillights, cameras, and a license plate incorporated with FC, followed by an image detection and beam-steering mechanism. In Section 3, to show the feasibility of the proposed system, a series of experimental analyses is presented, including the characterization of the FC-coupled license plate, investigation on the range of beam steering and tracking, and analysis on the communication performances. A real-time black-box video streaming through the beam-steering channel is demonstrated. Then, the discussions and conclusions are presented in Section 4.

## 2. Proposed Architecture

### 2.1. Proposed V2V-VLC System

**Figure 1:**
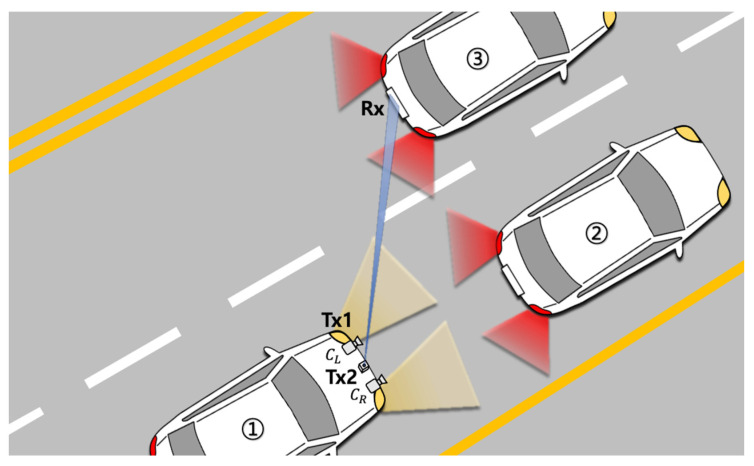
Illustration of the proposed V2V-VLC system.

Figure 1 shows an overall concept of the proposed V2V-VLC system. The vehicle ① uses two WLED headlamps as a transmitter and also has two front-view cameras ($C_{L}$ and $C_{R}$) to capture the taillights of the front vehicles (② and ③). The license plate located between the taillights plays a role as an optical receiver. It is composed of a rectangular FC and an avalanche photodetector (APD) located at the edge. Headlamps can be used for both lighting and data transmission purposes. A blue LD module is installed between the two cameras to deliver a high data-rate communication channel by beam steering. It also provides flexibility in reconfiguring the links for robustness against blockage and misalignment. The large coverage area of the FC leads to higher optical gain, which is suitable for both the headlamp and blue-LD-based communication.

### 2.2. License Plate Incorporated with Fluorescent Concentrator

**Figure 2:**
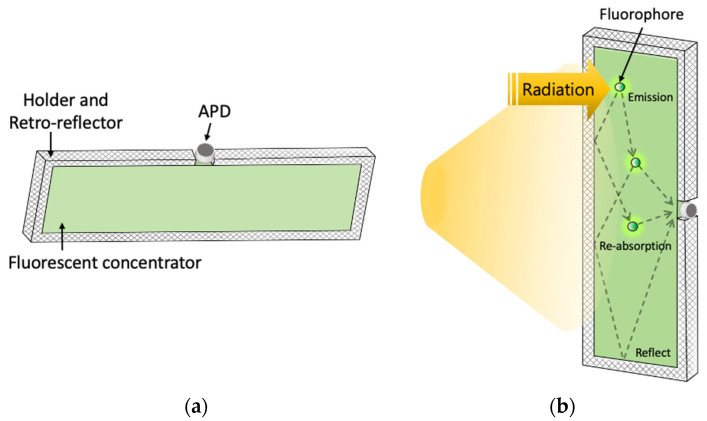
(**a**) Design of license-plate-shaped receiver; (**b**) schematic diagram of the physical process of a fluorescent concentrator.

Figure 2a shows the license-plate-shaped semi-transparent FC with an APD on the top. A holder is designed to fix the FC, and retro-reflectors are attached inside. The retro-reflectors direct the light efficiently back to the FC. Thus, high intensity (high optical gain) is provided from the small open area where the APD is placed.

As shown in Figure 2b, a portion of the absorbed light is converted to a longer wavelength light by Stoke shift from the fluorophores and re-emitted omni-directionally. The FC used in this work has the absorption peak at 450 nm. The majority of the converted light is guided to a narrow edge through the total internal reflection. A license plate is located at the backside of the semi-transparent FC. Since the license plate also reflects a portion of the incident light back to the FC, overall optical gain can be enhanced by a factor of 2.5 [14].

### 2.3. Proof-of-Concept Demonstration

**Figure 3:**
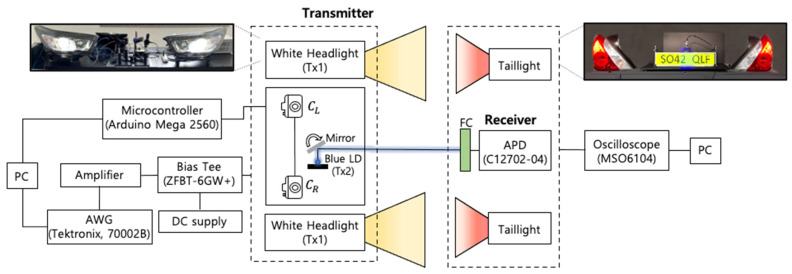
Schematic block diagram and experimental setup of the proposed V2V-VLC system.

The experimental setup for the feasibility test of the proposed system is illustrated in Figure 3. DCO-OFDM signal is generated by an arbitrary waveform generator (Tektronix, 70002B) and adjusted by an amplifier (Mini-Circuits, LZY-22+ and ZPUL-30P+). A bias-T (Mini-circuit, ZFBT-6GW+) is used to combine a suitable DC offset to ensure the positive polarity from the bipolar signal created by the AWG. Two types of transmitters are implemented: one with headlamps using high-power WLEDs and the other with a blue LD. The APD (Hamamatsu, C12702-04) at the receiver module detects the optical signal concentrated through the FC. Then, the corresponding electrical signal is captured by an oscilloscope (Keysight, MSO6104), followed by offline signal processing via MATLAB for communication performance analysis.

Next, two LED taillights (used as beacons) provide location information to two cameras (Pixy2) at the transmitter module. The information is used for beam steering and tracking. The steering angle is calculated by the values given from a microcontroller (Arduino Mega 2560) connected to the cameras. It also controls the steerable mirror (Optotune, MR-E-2) accordingly.

### 2.4. Beam-Steering Mechanism

**Figure 4:**
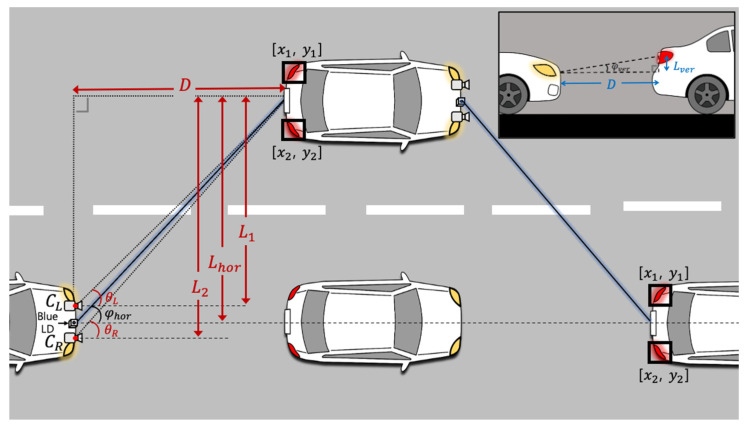
Geometric configuration for top and side views.

As shown in Figure 4, two cameras used in this experiment detect the taillights as objects, using the pre-registered features. Unlike the traditional visible light positioning systems based on trilateration or triangulation from separable light sources, the tail-lamp tracking method used in this work utilizes the disparity between images from the two cameras, constructing a type of stereo vision. The blue LD beam is steered to the FC-coupled license plate located in the middle of the two taillights. The relative location of the license plate is calculated in real time by applying the following equations:

(1) $$\left[x_{L}=\frac{x_{L1}+x_{L2}}{2},\;\;\;\;y_{L}=\frac{y_{L1}+y_{L2}}{2}\right]\;\left[x_{R}=\frac{x_{R1}+x_{R2}}{2},\;\;\;\;y_{R}=\frac{y_{R1}+y_{R2}}{2}\right]$$

There are two pairs of location information representing the relative position of the taillights seen from each camera. The microcontroller connected to camera sends the information to the PC via a serial port. $x_{L1}$, $x_{L2}$, $y_{L1}$, and $y_{L2}$ in Equation (1) mean the horizontal and vertical locations of the two taillights seen from the left camera, respectively. Likewise, $x_{R1}$, $x_{R2}$, $y_{R1}$, and $y_{R2}$ indicate such information seen from the right camera, respectively. Here, $x_{L}$, $y_{L}$, $x_{R}$, and $y_{R}$ denote the calculated horizontal and vertical location of the license plate using the information from the left and right camera. Using this information and Equation (2), the relative distances and angles from each camera ($L_{1}$, $L_{2}$, $\theta_{L}$ and $\theta_{R}$), as well as the perpendicular distance (*D*) described in Figure 4, can be calculated [13].

(2) $$D=L_{1}\cdot tan\left(\frac{\pi }{2}-\theta_{L}\right)=\;L_{2}\cdot tat\left(\frac{\pi }{2}-\theta_{R}\right)$$

(3) $$\varphi_{hor}={tan}^{-1}\left(\frac{L_{hor}}{D}\right)$$

(4) $$\varphi_{ver}={tan}^{-1}\left(\frac{L_{ver}}{D}\right)$$

Therefore, the horizontal angle ($\varphi_{hor})$ and vertical angle ($\varphi_{ver})$ between the receiver and blue LD module can be calculated by Equations (3) and (4), where $\varphi_{hor}$ and $\varphi_{ver}$ are the values that are ultimately used to adjust the angle of the steering mirror. The mirror also has a horizontal and a vertical axis that can be adjusted up to 50 degrees in each direction. The beam-steering module can control the beam to the target with tracking information updated in real time. This method can be applied to the vehicle in front and other neighboring vehicles.

## 3. Experiment Result

### 3.1. Analysis of Proposed FC-Coupled Number Plate

**Figure 5:**
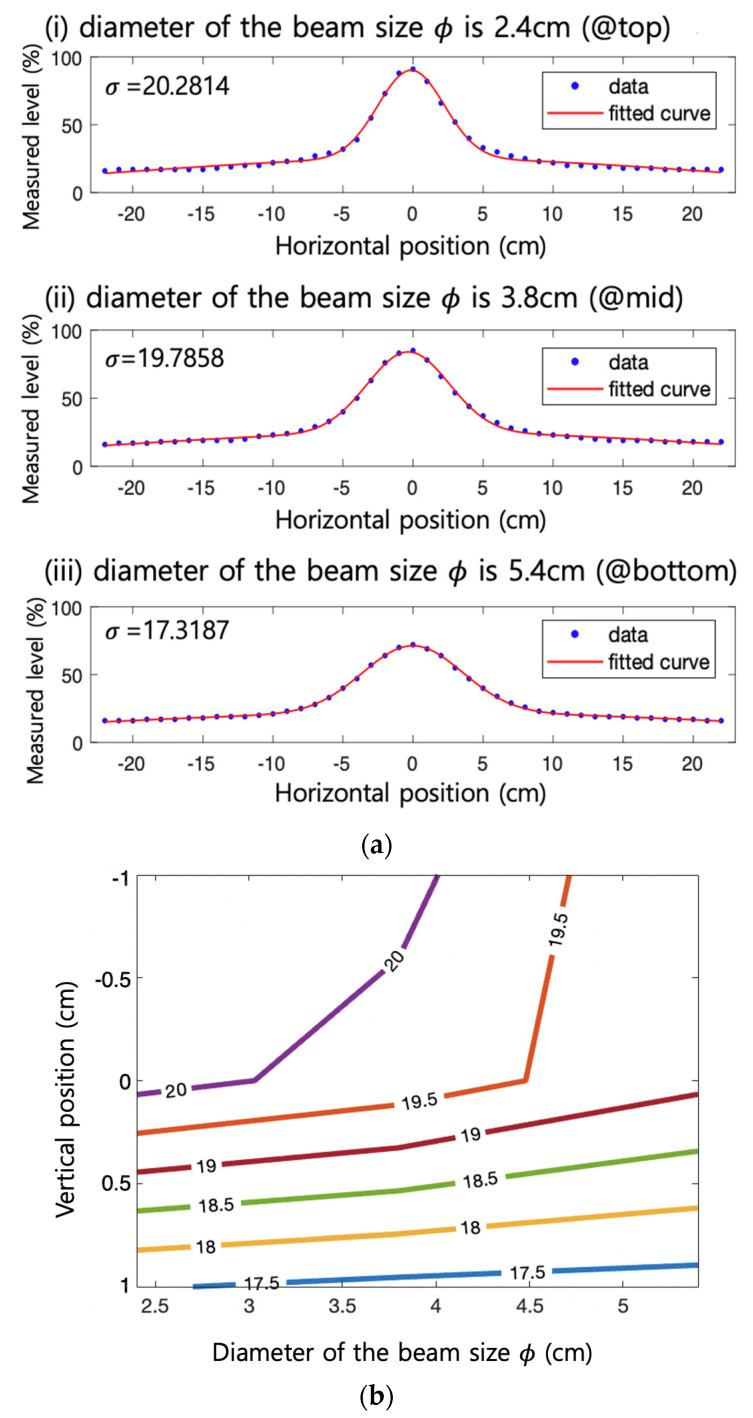
(**a**) Measured signal strength distribution and Gaussian fit; (**b**) overall standard deviation analysis.

An intensity analysis according to the beam size and incident light position is performed to characterize the influence of the incident beam pattern on the FC-coupled number plate. As shown in Figure 5a, the signal intensity distribution from the blue LD beam (Thorlabs, PL450B) is measured. DC and sinusoidal signals are adjusted to be in the linear driving condition and applied to the blue LD, and the beam diameter (*ϕ*) is varied to 2.4 cm, 3.8 cm, and 5.4 cm. The three locations (top, middle, and bottom) of the FC are measured separately. At the top, an APD is located to detect the signal. The three locations mean the vertical distance from the APD by 3 cm, 5.5 cm, 8 cm, respectively. The received sinusoidal peak to peak is measured, along with the length, by moving the horizontal position of the incident light with 1 cm interval. The value of 0 for the horizontal position means the center of the concentrator.

Case (i) in Figure 5a shows the received signal strength distribution when the smallest beam is incident on the top surface of the FC. Case (ii) is intermediate in terms of the beam location and the size, and Case (iii) presents the result from the case with the largest beam size at the bottom. The dots in the graph are the measured points, and the fitted curve is from the Gaussian model. As shown in the graph, the Gaussian fitted curves are in good agreement with data points. The standard deviation for each case is 17, 19.8, and 20.3, respectively. The overall standard deviation analysis from a series of similar measurements is shown in Figure 5b. In the y-axis of the graph, 1, 0, and −1 mean the top, middle, and bottom (which means the farthest point from the APD) of the FC, respectively. As a result, it is confirmed that the shorter the distance from the receiver, the higher the variance of the channel, and the larger the spot diameter, the lower the variance.

**Figure 6:**
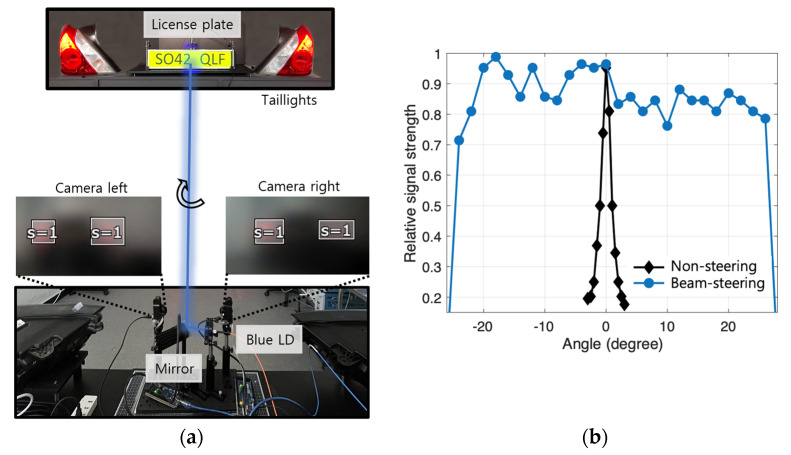
(**a**) Comparison of received signal strength with and without beam steering; (**b**) Beam-steering and tracking setup.

Next, the feasibility of the beam steering method introduced in Section 2.4 is investigated at 5 m link distance. Figure 6a shows the experimental setup. First, the taillights seen from the left and the right camera are tracked as an object that is indicated as ‘S=1′ in the figure. Using the differential location of the tracked objects, the relative position of the license plate is calculated, which is the information used for steering the mirror. Changing the angle of the transmitter module, the relative signal strength by applying the beam-steering and tracking method is monitored. As shown in Figure 6b, without the beam-steering method (‘Non-steering’), the link is abruptly broken as soon as the beam is off the license plate. Consequently, at an angle beyond +−3 degrees, there is no signal detected by APD. However, when activating the beam-steering method, the operational angle is much improved. The maximum beam-steering angle is measured up to +−25 degrees. The camera used in this work has a frame rate of 60 frame/s and resolution of 1296 × 976; the mirror has an optomechanical movement speed of 0.65 ms/degree; and the computational time is sub-milliseconds. Consequently, the worst-case latency stemming from 50 degrees beam steering (max. steerable angle of the system) is calculated to be ~50 ms.

### 3.2. Communication Test

**Figure 7:**
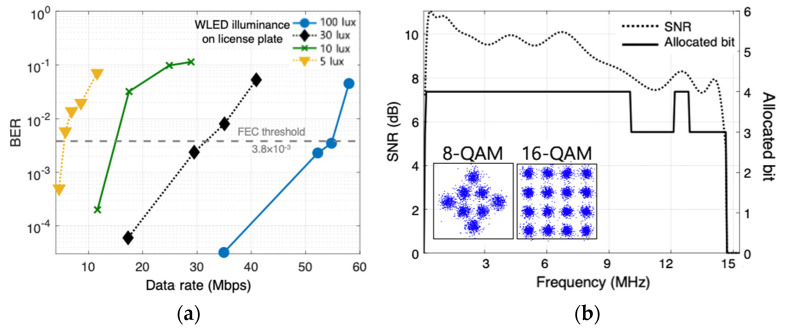
(**a**) BER performance according to different lux levels of LED headlamp; and (**b**) constellations, measured SNR and allocated bits with adaptive bit and power loading.

Firstly, bit error rate (BER) performance is tested by varying the illuminance (lux) on the license plate from the two WLED-based headlamps. The illuminance is closely linked with the actual link distance on the road. For instance, the levels used in this work are 5 lux, 10 lux, 30 lux, and 100 lux, and they represent the distances of about 80 m, 60 m, 30 m, and 15 m, respectively [6,15]. The commercial WLED headlamps are modulated with a DCO-OFDM using an adaptive bit and power-loading scheme. The two WLED modules are synchronized and send the same data stream. A detailed description of the modulation technique used here is found in Ref. [16]. The FFT size and cyclic prefix length were set to 512 and 10, respectively. To ensure a reliable experiment, the uniform flood light covering the entire license plate is applied. The measured data rate versus BER plots are shown in Figure 7a.

Even at 5 lux illuminance, the data rate of 4.5 Mbps under a forward error correction (FEC) with BER threshold of 3.8 × 10^−3^ is achieved. At 100 lux, the data rate is much improved to 54 Mbps. This is mainly due to the high SNR available from the high illuminance. The received 8 QAM and 16 QAM constellations for the data rate of 52 Mbps at 100 lux case are presented in Figure 7b. The SNR is measured by analyzing the received signal statistically in comparison with the transmitted signal [16]. Additionally, shown are the measured SNR and the allocated bits, accordingly.

**Figure 8:**
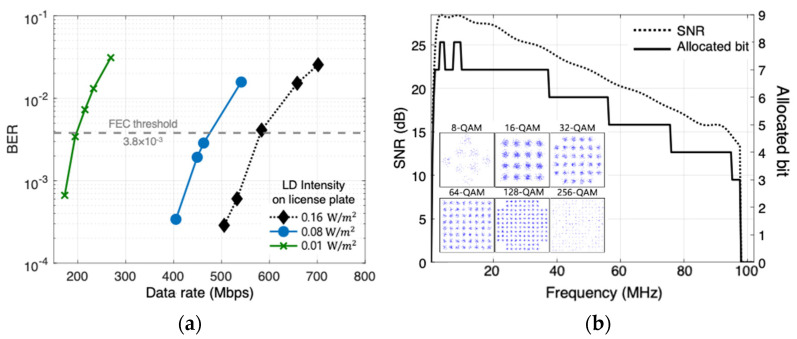
(**a**) BER performance according to output power of LD; and (**b**) constellations, measured SNR and allocated bits with adaptive bit and power loading.

The experiment is also performed for the blue beam-steering channel. The intensity on the license plate is varied by adjusting the driving conditions and monitored by optical power meter (Thorlabs, PM400). The tested intensities on the license plate are 0.01, 0.08, and 0.16 $W/m^{2}$. As shown in Figure 8a, the data rates of 194 Mbps, 462 Mbps, and 532 Mbps are achieved, respectively. Additionally, the constellations, the measured SNR, and the allocated bits for the case of 532 Mbps are shown in Figure 8b.

**Figure 9:**
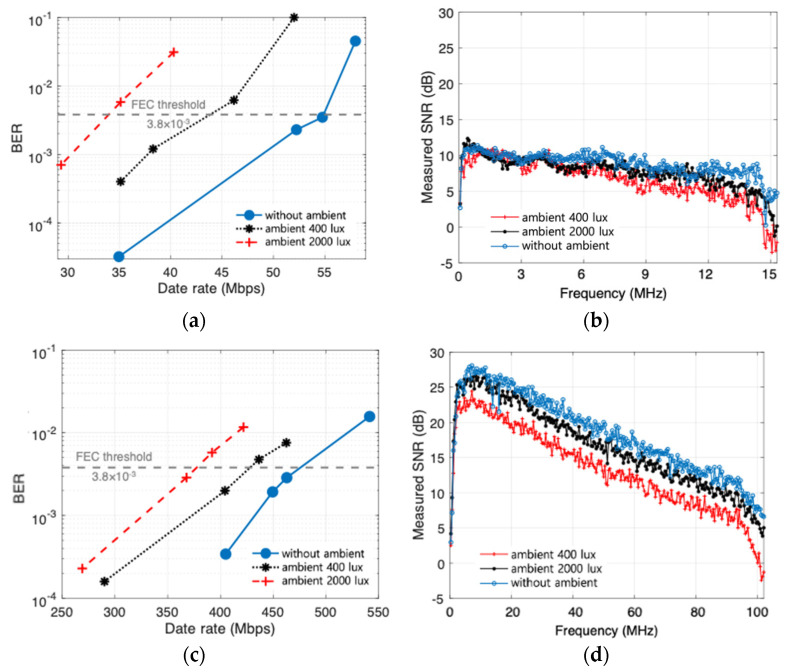
BER performance under ambient light for (**a**) headlamp at 100 lux and (**b**) measured SNR, and (**c**) BER performance under ambient light for blue LD beam at 0.08 $W/m^{2}$ and (**d**) measured SNR.

Next, experiments to check the impact of ambient light on communication performance are conducted. A high-power WLED is used to consider a strong ambient light from adjacent light sources. The ambient light is applied to both the headlamp channel and the blue-beam channel. Levels of 400 lux and 2000 lux are investigated, and the case without the ambient light is also presented as a reference. As shown in Figure 9a, in the case of headlamp channel, the data rate without the ambient light is 54 Mbps with 100 lux. However, the rate is decreased to 38 Mbps and 29.3 Mbps under the ambient light with 400 lux and 2000 lux, respectively, as shown in Figure 9b. Additionally, the data rate of 462 Mbps achieved with 0.08 $W/m^{2}$ from the blue LD channel is decreased to 404 Mbps and 367 Mbps under the ambient light with 400 lux and 2000 lux, respectively. The effect of ambient light on the SNR in each case is shown in Figure 9b,d. Although the SNR deteriorated due to the ambient light much more strongly than the communication signal, the proposed system still has a reasonably good performance. This is due to the fact that the APD placed downward in the proposed system does not see the ambient light directly incident on the license plate. Additionally, the FC in front of it only absorbs a certain portion of the incoming ambient light by its wavelength-dependent absorption property [11].

### 3.3. Demonstration of Real-Time Video Transmission

**Figure 10:**
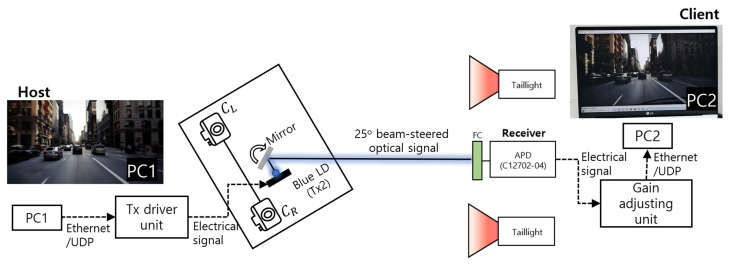
Real-time video-streaming demonstration.

A real-time video streaming considering the case of streaming a black-box video via V2V-VLC is demonstrated. Here, the blue beam-steering channel is used. As shown in Figure 10, two PCs work as a streaming host and a client. The host PC streams a real-time video through the Ethernet. In this demonstration, user datagram protocol (UDP) is applied, and through the Tx driver unit, the signal is conditioned and applied to the LD transmitter. Following the beam-steering and tracking mechanism introduced in Section 2.4, the data are transmitted to the FC-coupled license plate. The received signal is converted back to the Ethernet signal through the gain adjusting unit, subsequently being played at the client PC. To test the robustness of the proposed system, a demonstration is conducted with a beam-steering angle of 25 degrees, also under an ambient light level of 2000 lux. Even with these conditions, as shown in the figure, real-time black-box video streaming is successfully demonstrated.

## 4. Discussions and Conclusions

In this work, we suggested FC-coupled license plate with a beam-steering-technique-based V2V-VLC system. The feature of FC was tested and confirmed that there are advantages, such as high gain and wide FoV. Off-the-shelf white WLED headlamps and a blue LD channel to the FC-coupled license plate were evaluated with DCO-OFDM scheme, under various practical illuminances. As a solution to tackle situations such as LOS blockage and misalignment, the beam-steering and tracking system using the image processing and the steerable mirror was employed. The maximum data rates achieved in this demonstration were 54 Mbps and 532 Mbps from the WLED headlamps and from the blue LD, respectively. In particular, the blue LD was able to configure a beam-steering and tracking system with ±25°. In addition, a real-time black-box video streaming through the beam-steering channel was demonstrated.

Future work includes constructing a bi-directional V2V-VLC link using the modulated taillights through optical camera communications, the investigation on the dynamic outdoor environment, including the signal crosstalk caused by the modulated beams from adjacent vehicles, developing a multi-vehicle tracking and selection mechanism. Further optomechanical optimization will also be performed for practical and compact form factor.

## Data Availability

The data presented in this study are available on request from the corresponding author and on his personal website.
